# Correction: Stress in autism (STREAM): A study protocol on the role of circadian activity, sleep quality and sensory reactivity

**DOI:** 10.1371/journal.pone.0329798

**Published:** 2025-08-05

**Authors:** Clara C. Gernert, Christine M. Falter-Wagner, Valdas Noreika, Barbara Jachs, Nazia Jassim, Kathryn Gibbs, Joaquim Streicher, Hannah Betts, Tristan A. Bekinschtein

The authors are listed out of order. Please view the correct author order, affiliations, and citation here:

Christine M. Falter-Wagner^1^, Clara C. Gernert^1,2^, Valdas Noreika^2,3^, Barbara Jachs^2^,Nazia Jassim^4,5,^ Kathryn Gibbs^6^, Joaquim Streicher^2^, Hannah Betts^2^, Tristan A. BekinschteinID^2^

**1** Department of Psychiatry and Psychotherapy, LMU University Hospital, LMU Munich, Munich, Germany, **2** Consciousness and Cognition Lab, Department of Psychology, University of Cambridge, Cambridge, United Kingdom, **3** Department of Psychology, School of Biological and Behavioural Sciences, Queen Mary University of London, London, United Kingdom, **4** Autism Research Centre, Department of Psychiatry, University of Cambridge, Cambridge, United Kingdom, **5** Prediction and Learning Lab, Department of Psychology, University of Cambridge, Cambridge, United Kingdom, **6** Independent researcher

Falter-Wagner CM, Gernert CC, Noreika V, Jachs B, Jassim N, Gibbs K, et al. (2024) Stress in autism (STREAM): A study protocol on the role of circadian activity, sleep quality and sensory reactivity. PLoS ONE 19(5): e0303209. https://doi.org/10.1371/journal.pone.0303209
